# Neonatal nursing led research activity in the UK: a survey of current practice

**DOI:** 10.1186/s12912-021-00719-8

**Published:** 2021-10-18

**Authors:** Katie Gallagher, Julia Petty, Joanne Cooper, Neil Marlow

**Affiliations:** 1 Elizabeth Garrett Anderson Institute for Women’s Health, University College London, London, WC1E 6AU UK; 2 School of Health and Social Work, The University of Hertfordshire, Hatfield, UK; 3 Nottingham University Hospitals NHS Trust, Institute of Care Excellence, Derwent House, City Campus, Hucknall Road, Nottingham, NG5 1PB UK

**Keywords:** Neonatal nursing, Nursing research, Nursing education, Clinical academic careers

## Abstract

**Background:**

Neonatal nurses are ideally placed in practice to undertake research enhancing the care of families. More information is required, however, around neonatal nursing led research to advance leadership in this area. The aim of this study was to determine neonatal nursing led research activity within the UK.

**Methods:**

The study used a web-based survey design and neonatal nurses were eligible if they were working at or towards Masters or Doctoral level qualification in the UK. The survey was distributed to members of the Neonatal Nurses Association, UK Schools of Nursing and shared on social media pages of authors and professional organisations. Results were analysed using descriptive and frequency statistics and content analysis.

**Results:**

Of the 56 respondents, 14% (*n* = 8) had a Doctoral level qualification and 43% (*n* = 24) of participants held a Masters qualification. Lack of time and funding knowledge was the largest barrier to research. Only 30% (*n* = 3) of participants had a research mentor and only 18% (*n* = 3) were from a neonatal nursing background.

**Conclusions:**

There are limited numbers of neonatal nurses undertaking or leading nursing research in the UK. Further support is required to enhance clinical academic career trajectories to ensure research is a viable pathway for future generations of neonatal nurses.

**Supplementary Information:**

The online version contains supplementary material available at 10.1186/s12912-021-00719-8.

## Background

Engagement with clinical research at National Health Service (NHS) organisation level is associated with improved Trust performance, reduced mortality rates and improved outcomes for both patients and staff [1–3]. Nurses are ideally placed to progress this research from the bedside, with the ability to respond to the rapid proliferation of clinical questions which can advance patient centred care [4, 5]. As nursing advances accordingly, this necessitates nurse researchers who can effectively lead research programmes in practice [5]. The neonatal nursing specialty serves as a prime example of advancing roles and development, where research is key to enhancing outcomes of sick and premature babies and their families.

Over the past decade in the UK various strategies have been developed to support the advancement of Nurses, Midwives and Allied Health Professionals (NMAHP) in research leadership [6–9]. These have focused resource on appropriate research training for nurses to drive and lead research underpinning practice as part of Clinical Academic Career pathways. Higher Education England (HEE) and the National Institute of Health Research (NIHR) have developed dedicated NMAHP funding schemes to support these pathways, although evaluation suggests nursing uptake remains low in comparison to AHP counterparts and individual experiences are varied [10, 11]. The Royal College of Nursing published career pathway guidance for neonatal nurses in 2015 [12]. This complex speciality, encompassing around 5000 nurses in 196 neonatal units across the UK [13, 14] represents an area of huge potential for nursing led research programmes to transform experiences and outcomes for infants, families and staff. Recommendations suggested a minimum of Masters level education when working as an expert practitioner, with research active neonatal nurses identifying, participating and leading research projects to contribute to the overall advancement of neonatal practice [12].

Despite recommendations for the benefits of nursing leadership in research, it is estimated that only around 0.1% of the entire NMAHP workforce are clinical academics, as compared to nearly 5% of the medical consultant workforce [15, 16]. Even less is known about neonatal nursing research activity or how many nurses are engaged in clinical research in this area, outside of research roles which traditionally support the delivery of clinical trials commonly led by others. In order to advance the neonatal nursing research agenda, more information is required relating to the number and experiences of research active neonatal nurses in the UK. The aim of this study was therefore to determine neonatal nursing led research activity within the UK. To the best of our knowledge, this is the first study of its kind to identify the number of nurses working at or towards Masters or Doctoral level qualification, current roles in practice and barriers and facilitators in undertaking research.

## Methods

A web-based survey was developed to gather data on educational attainment, current roles, research activity and barriers and facilitators to research amongst neonatal nurses in the UK. The survey consisted of 17 questions comprising both multiple choice questions and open-ended responses. Multiple choice questions allowed participants to self-identify with, for example, their level of practice (Band, education level) and elements of their role (teaching, research, practice, management) with options to expand upon their responses and open-ended questions allowing participants to provide information around areas including research mentors, interests and activities. Content validity was ensured through expert review by experienced researchers in the neonatal field; the survey was also piloted with colleagues to ensure it was clear and easy to complete. Following the pilot we added an open ended response to allow participants to identify the type of professional Doctorate being studied to allow for different pathways (i.e education, health research). Ethical approval was gained from the University College London Research Ethics Committee (REC ID: 16059/002) and the University of Hertfordshire Research Ethics Committee (REC ID: 000009 Petty747825).

### Participants and setting

Neonatal nurses were eligible to complete the questionnaire if they had worked or were working towards Masters or Doctoral level qualification in the UK.

### Data collection

A web-based survey was designed in UCL Opinio, hosted by UCL Information Services Divisions (ISD) infrastructure and meeting ISD standards. The Participant Information Sheet was provided at the start of the survey, followed by the option to sign a digital informed consent; consent was sought for participants who wished to share their email address and form part of a new neonatal nursing research group to develop collaborations and networks amongst peers. Email details were not required otherwise and so presumed consent was accepted upon participant completion of the survey. In an attempt to reach as many neonatal nurses as possible working in either clinical or academic positions, an email containing a brief outline of the study and the survey link was sent to all members of the UK Neonatal Nurses Association (NNA), the NNA education subgroup LEARN, and to all Schools of Nursing in the UK requesting further distribution as appropriate. A link to the survey was also posted on the pages of relevant social media sites (Facebook™, Twitter and LinkedIn) of the authors and of relevant professional organisations, including the Neonatal Nurse Association and the Royal College of Nursing: Children’s and Young People’s Group.

### Data analysis

Data was downloaded from Opinio directly into Microsoft Excel for analysis. Descriptive statistics were used to analyse demographic data and frequency statistics used to analyse multiple choice questions. Content analysis was used to analyse data from open ended questions. This method is useful when conducting exploratory work in a relatively unknown area, as it allows for the distilling of words (text) into fewer content related themes and provides a useful way to quantify the data through counts of these issues [17, 18]. Data was read and re read to get a sense of immersion, with initial coding undertaken by KG to recognise basic concepts or ‘themes’ within the text which reflected the research questions [17]. Frequency counts were then assigned to each concept to determine the commonality of the responses, with text used as illustration. Categories were discussed with a second researcher (JP) to optimise rigour and trustworthiness of the qualitative component of the data [19].

## Results

**Fig. 1:**
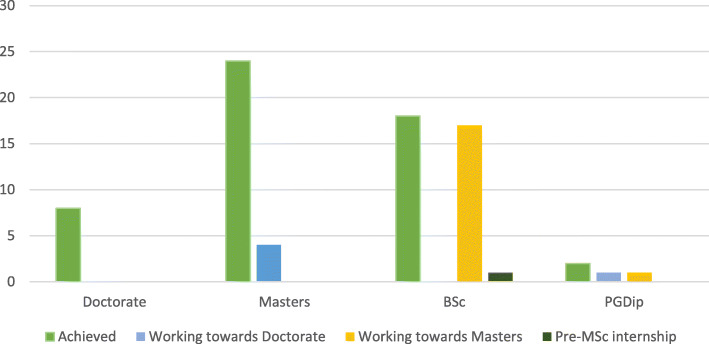
Neonatal Nurses educational attainment: achieved and working towards

Fifty-seven neonatal nurses completed the survey. One participant had trained in and was working overseas, so was excluded from the study. A total of 56 responses were therefore included in the analysis. The majority of respondents were female (96%; *n* = 54). The mean number of years working as a neonatal nurse was 16 years (range: 2 to 32 years). Of the 56 respondents, 14% (*n* = 8) had a Doctoral level qualification (5 PhDs and 3 Professional Doctorates). A further 9% (*n* = 5) were registered on a Doctoral level programme (3 PhD and 2 Professional Doctorates). Forty-three percent (*n* = 24) had a Masters level qualification, with a further 32% (18) registered on a Masters programme. One participant was registered on an NIHR pre-Masters internship (Fig. 1).

### Doctoral level qualification

Of the 11 participants either with or working towards a Doctoral level qualification, 64% (*n* = 7) had funding from a fellowship (*n* = 1), stipend (*n* = 2), charity (*n* = 2), professional body (*n* = 1) or as part of a larger study (*n* = 1). Of the 8 participants who had received their Doctoral qualification, 3 were working at Agenda for Change (AfC) Band 8 level, 1 at Band 7, 1 as an Advanced Neonatal Nurse Practitioner (ANNP) at registrar level, 1 at Band 6 and 2 in Higher Education Institutions (HEI). Study participants’ main role comprised responsibility for teaching plus either research or management (*n* = 3), clinical practice (*n* = 2), research (*n* = 1), research and management (*n* = 1). One participant reported working concurrently in clinical practice and academia. Dedicated research time varied from 0, 5, 25, 40%, (*n* = 1 respectively) to allocated days per year (*n* = 1), variable (*n* = 1) and 100% (*n* = 2).

### Masters level qualification

**Fig. 2:**
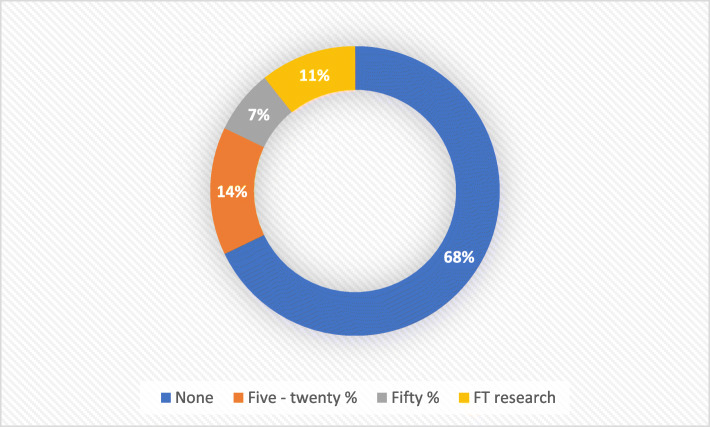
Reported % of work role time dedicated to research for Masters level qualified neonatal nurses

Of the 42 participants having attained or working towards Masters level qualification, 40% (*n* = 17) reported receiving funding through their NHS Trust (*n* = 9), separate research funding (*n* = 3), educational initiative (*n* = 2), internship/secondment (*n* = 2) or the apprenticeship levy (*n* = 1). The majority of participants who had received their Masters level qualification (*n* = 28) were working at AfC Band 7 level or above. Nearly a third (32% *n* = 9) were working in clinical practice, including at ANNP level. A further 29% worked in teaching roles (*n* = 8) and 21% (*n* = 6) in a mixture of teaching, research, clinical practice and management roles. The remaining 5 participants worked in management roles (*n* = 2), research (*n* = 1), HEI (*n* = 1) or were undertaking a secondment (*n* = 1). The majority of participants working with a Masters level qualification reported no or minimal dedicated and protected research time (Fig. 2).

### Research support

Participants were asked to report if regular nursing research activities such as journal clubs were undertaken on their unit and what these involved. There were 53 responses; 45% (*n* = 24) said that these were regular activities, 40% (*n* = 21) said there were none and 15% (*n* = 3) worked outside of clinical practice. For those responding yes, 92% (*n* = 22) reported that the most popular research activity reported was journal clubs (77% *n* = 17). Over a third of those reporting journal clubs, however, (41% *n* = 7) said that these were medically focused and/or led by medical colleagues. Other activities included research team meetings (*n* = 2), management team reviews, quality improvement work, active research teams and management team review of research activities (*n* = 1 respectively). Participants were asked if they either had or were personally involved in any nursing research in either clinical or academic work, including their area of research interest. Fifty-one participants responded, with 98% (*n* = 50) reporting activity as part of Doctoral and Masters level dissertations, post-doctoral work and clinical audits and service evaluations. The main themes of research interest were parental involvement in care (*n* = 12), pharmacological research (*n* = 10), education (*n* = 9), outcomes (*n* = 4), developmental care (*n* = 3), service provision (*n* = 3), respiratory (*n* = 3) feeding issues (*n* = 2), end of life care (*n* = 2), ethico-legal issues (*n* = 1) and skin (*n* = 1). (Table 1).

**Table 1:** Reported research areas of neonatal nurses

Research Theme	Research area (participants interested / working in this area)
Parental involvement	Comfort care, experiences during stay (3), decision making, post discharge complex care, family involvement/impact (3), satisfaction, Kangaroo Mother Care (KMC), trial participation
Pharmacological	Neonatal Abstinence Syndrome (NAS) (2), pain (2), morphine, near misses, antibiotic use (2), Selective Serotonin Reuptake Inhibitors (SSRI), hearing loss
Education	Network delivered preceptorship, skills training, framework evaluation, thermal control, learning opportunities, experiences of (nurses: 1 / students: 1), evidence implementation, leadership
Outcomes	Mental health, NAS, long term infant outcomes, staff impact on short term infant outcomes
Developmental care	Family Integrated Care (FICare), attitudes, quiet time
Service provision	Transitional care, postnatal sepsis screening, transport ventilation
Respiratory	Ventilation (2), lung function
Feeding issues	Orogastric tube (OGT) use, Gastro Oesophageal Reflux (GOR)
End-of-Life care	Palliative care, organ donation
Ethico-legal	Legal & ethical issues (2)
Skin	Tissue viability

Participants were asked if they had a research mentor and, if applicable, the background of their mentor. Only 30% (*n* = 17) replied yes, and of these only 18% (*n* = 3) of mentors were from a neonatal nursing background. The majority of mentors were medical consultants, non-nursing professionals, midwifery and child health academics.

### Barriers & Facilitators

**Fig. 3:**
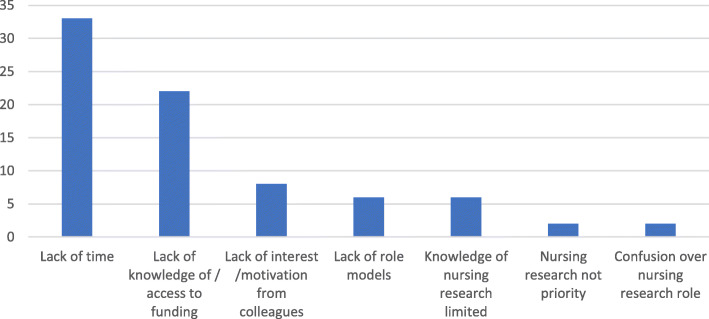
Participants perceived barriers to neonatal nursing led research

When asked about barriers to research, 89% (*n* = 50) of participants commented. Lack of time for research was perceived to be the largest barrier (*n* = 33), followed by lack of knowledge of or access to funding (*n* = 22). Limited support from colleagues (*n* = 8) and too few role models (*n* = 6) were also reported (Fig. 3). Participants were also asked about facilitators to nursing research; only 18% (*n* = 10) responded to this question. The majority of the respondents (70% *n* = 7) stated that support was provided by a facilitator, with 20% (*n* = 2) stating a positive research culture in the clinical environment and a further participant stating their role as clinical research nurse facilitated research. Comments to support the findings from the content analysis can be found in supplementary Table 1.

Participants were asked to share other comments they had regarding the role of higher degrees in neonatal nursing, and the role of neonatal nurses in research. There were 25 responses (45%) and of these, 32% (*n* = 8) raised the importance of establishing advanced research career pathways for neonatal nurses outside of the traditional ANNP and management roles. A further 28% (*n* = 7) stated the ‘essential’ nature of embedding research in nursing education and 12% (*n* = 3) stated the importance of role modelling in neonatal nursing research.

Finally, participants were asked whether they would like to form a new network of neonatal nurses involved in collaboration, and to share their rationale for their choice. Out of 30 responses to this question, 26 responded yes for reasons including collaboration (37% *n* = 11), to raise the profile of neonatal nursing (37% *n* = 11), personal development (12% *n* = 3) and to increase future neonatal nursing mentors (4% *n* = 1). Four participants responded no, stating lack of time (*n* = 3) experience and expertise (*n* = 1).

## Discussion

The aim of this study was to identify and describe neonatal nursing research activity within the UK. The study is limited by its response rate as there may be neonatal nurses practising at Doctoral or Masters level who did not complete the survey. The survey was advertised widely on social media and through all schools of nursing in an attempt to reach as many neonatal nurses as possible, however it is acknowledged that this may have impacted on the findings.

Our findings indicate that, from this albeit limited sample, there are 8 neonatal nurses currently working at or above Doctoral level. Including the authors of this paper, this number increases to 10. Of the estimated 5000 neonatal nurses in today’s NHS [14] this equates to 0.2% of the current workforce. When considering that only 1 participant reported active roles in both academic and clinical practice, the clinical academic rate for neonatal nurses in the UK is 0.02%. This is unsurprising when considering that there are currently no professors of neonatal nursing in the UK, although a clinical element is not a prerequisite for Professorship in this country. Following survey completion, one participant was recently made an associate professor, despite a small number of children’s and specialist nursing professors [20].

Like previous studies, our findings indicate that there are multiple barriers in clinical practice to undertaking nursing led research. HEE conducted a review of clinical academic careers in 2017 and found the availability of funding, time, positions and inadequate support from employing intuitions were all barriers to progressing research related careers in non-medical health professionals [21]. The report also highlighted the difficulties faced when transitioning to a post-Doctoral phase of research, citing that the majority of participants went into either an academic or clinical post with no formal research sessions. This is again reflective of our findings, which indicate that participants working at Doctoral level do not have a structured post-doctoral career pathway which incorporates their research skills within a clinical academic role, instead assuming a variety of traditional roles within and across both academia and practice. A study exploring the experiences of NMAHPs pursuing clinical academic careers in the East Midlands recently found that participants likened the career pathway to a pyramid, with research progression challenging and poor financial infrastructure to support career choices post-Doctoral qualification [22]. These findings indicate an urgent need for defined career trajectories outside of the traditional management or ANNP pathway, career support and associated pay scales for clinical academic neonatal nursing researchers. This would contribute to developing an innovative workforce who feel their contribution is valued and who are able to continue to pursue their passion of improving infant and family care and outcomes through research [11, 22–24].

In an attempt to address the limited opportunities in practice, a number of UK Institutions are collaborating with NHS organisations to identify research priority areas and develop defined NMAHP clinical academic pathways [23, 25]. The Council of Deans Clinical Academic Roles Implementation Network (CARIN) provides support to promote and evaluate these joint clinical academic roles, along with advice on how to develop and sustain research activity [26, 27]. At the heart of these collaborations are the identification and recruitment of high potential individuals who can establish successful funding revenue which can transform practice. In our study we have highlighted a number of participants across the UK who are currently working towards Masters and Doctoral level qualification in neonatal nursing, who could benefit from such an approach and mentorship from experienced clinicians. The importance of such organisational support cannot be underestimated; research mentors and institutional leadership, identified as barriers in our own study, have been found to be the highest facilitator for nursing research and the primary barrier when absent [21, 28, 29]. In our study only 30% (*n* = 17) of participants had a mentor, and only 3 mentors were from a neonatal nursing background. Addressing this issue through the development of research active neonatal nurses will allow us to build a network of mentors who can provide appropriate support for early, mid and senior career level researchers and support the advancement of nurse led research and career progression.

To centralise nursing research and support in the wider organisational agenda, a small number of NHS organisations are pursuing American Nurses Credentialing Centre (ANCC) Magnet® accreditation, an ANCC initiative where recognised hospitals have successfully aligned education, infrastructure and resources to support nursing excellence (incorporating research) in clinical settings [30, 31]. Nottingham University Hospitals NHS Trust were designated ANCC Magnet® (Nottingham City Campus) and Pathway to Excellence® (Nottingham Children’s Hospital) accredited in 2020, representing the only dual accredited organisation in Europe. Evaluation of outcomes in Magnet® designated hospitals have shown higher levels of job satisfaction and less reported burnout in nurses along with improved patient outcomes [32, 33]. A core domain of nursing excellence within Magnet® accreditation relates to New Knowledge, Innovation and Improvement, thereby focusing attention on nursing research cultures, infrastructure and evidence based practice. Evidence outside of the United States (US) remains limited however, and high quality research is required to measure the impact of hospital accreditation [34]. Accordingly, a grant from the European Union was recently awarded to an international team of researchers to explore the feasibility and sustainability study of the Magnet® model in over 60 hospitals from Europe [35]. Results from this study may prove pivotal for nursing clinical academic careers in the future.

The ability of neonatal nurses to contribute, develop and lead research which shapes clinical practice remains of pivotal importance. There is a national deficit of an estimated 40,000 nurses in the UK with particular challenges in neonatal nursing, allowing nurses the flexibility to work where they wish; recruitment strategies should therefore draw on the opportunities afforded by clinical academic role development [36]. Developing innovative, defined, financially supported clinical academic pathways may be one way of promoting staff loyalty and excellence through empowerment and autonomy, attracting and retaining high quality staff [11, 22, 23, 37]. One of the biggest barriers identified in this study was lack of time to undertake research; participants’ comments and reports of limited or absent time allocated to research activities in their role highlighted this issue. In the US, several authors have addressed this issue by demonstrating the Return On Investment (ROI) of PhD qualified nurses in clinical practice, highlighting total overall organisational savings of up to $9 million following evidence based nurse led interventions improving patient outcomes in areas such as pressure ulcers and chest tube site dressings [38, 39]. In the UK, studies have again highlighted the impact of nurse led interventions in areas such as less inappropriate use of antibiotics, reduced length of stay in patients in mental health crisis and post anaesthesia dementia care [23, 40]. Highlighting the impact that research active nurses can have in practice could be a huge step in achieving wider organisational support for specialised clinical academic career pathways. In neonatal nursing various studies have identified nursing research priorities both globally and within Europe; the research interests of participants in this study are all reflected in these studies highlighting the potential to develop networks of enthusiastic, motivated neonatal nursing led research teams who can highlight the impact of evidence-based interventions within the UK in areas including staff education, parental involvement, medical errors and end-of-life care [41, 42]. At a time when organisational resources in academic and clinical practice resources are low, strategies such as professional networking, collaboration with outside institutions and, for those teaching in Institutions, working with students (and their mentors in practice) to align similar research interests to facilitate research, must be encouraged to allow nurses to engage with research [43, 44]. There is huge scope for neonatal nurse led research which could transform experiences and outcomes of families, and we must encourage and support nurses to embrace these opportunities.

The results of this study have implications for the future of the neonatal nursing workforce. Much more work is required to develop and initiate opportunities for neonatal nurses who are passionate about research to encourage clinical academic careers in this speciality. At Institutional and organisation level, collaboration is required to develop and evaluate these roles for neonatal nurses at all levels, to ensure that we can attract high-talent nurses early in their career and maximise their research career potential. Development of transition funding to support immediate post-doctoral research nurses in clinical practice would allow for neonatal nurses to continue to build a programme of research post-Doctoral level qualification. At researcher level, there are recommended measures we can and will take to facilitate and initiate networks and collaboration:

- Development of a new network of research active neonatal nurses to forge collaboration and develop research proposals
- Harnessing social media to engage neonatal nurses in current nursing led neonatal research and develop networks both within the UK and abroad
- Development of informal network neonatal nurse mentors to facilitate and guide junior and mid-career researchers
- Centralisation of neonatal nursing research information online, including projects currently being undertaken, sample research project forms, funding opportunities, recently published papers

## Conclusion

Neonatal nursing is a highly specialised and complex area of care with much scope for nurse led research which could improve the experiences of infants and families. Despite this, there are a limited number of research active neonatal nurses in clinical practice with few opportunities to progress in this area. Development of role models and support to enhance clinical academic career trajectories are required to ensure that this pathway becomes a viable option for the future generation of neonatal nurses, allowing us to shape and influence the future of neonatal nursing care.

## Supplementary Information

**Additional file 1: Supplementary Table 1.** Participants perceived barriers and facilitators to research and interest in forming neonatal nursing specialist research group

## Data Availability

The datasets used and/or analysed during the current study are available from the corresponding author on reasonable request.

## References

[1] Ozdemir BA, Karthikesalingam A, Sinha S, Poloniecki JD, Hinchliffe RJ, Thompson MM (2015). Research activity and the association with mortality. PLoS One.

[2] Jonker L, Fisher SJ, Dagnan D (2020). Patients admitted to more research-active hospitals have more confidence in staff and are better informed about their condition and medication: results from a retrospective cross-sectional study. J Eval Clin Pract.

[3] Boaz A, Hanney S, Jones T, Soper B (2015). Does the engagement of clinicians and organisations in research improve healthcare performance: a three-stage review. BMJ Open.

[4] Grace PJ, Willis DG, Roy C (2016). Jones DA profession at the crossroads: a dialog concerning the preparation of nursing scholars and leaders. Nurs Outlook.

[5] Polomano RC, Giordano NA, Miyamoto S, Trautman D, Kempf S, Nuzzo PM (2020). Emerging roles for research intensive PhD prepared nurses: beyond faculty positions. J Prof Nurs.

[6] Department of Health (2012). Developing the role of the clinical academic researcher in the nursing, midwifery and allied health professionals.

[7] Willis (2015). Raising the bar. Shape of caring: A review of the future education and training of Registered Nurses and care assistants.

[8] NHS England (2016). Leading Chenge, Adding Value. A framework for nursing, midwifery and care staff.

[9] UKCRC (2007). Developing the best research professionals - qualified graduate nurses: recommendations for preparing and supporting clinical academic nurses of the future.

[10] Baltruks D, Callaghan P (2018). Nursing, midwifery and allied health clinical academic research careers in the UK.

[11] Cowley A, Diver C, Edgley A, Cooper J (2020). Capitalising on the transformational opportunities of early clinical academic career training for nurses, midwives and allied health professionals. BMC Med Educ.

[12] Royal College of Nursing (2015). Career, education and competence framework for neonatal nursing in the UK.

[13] Neonatal Transport Group (2020). UK neonatal units.

[14] National Health Service (2020). Roles in nursing: Neonatal nurse.

[15] Medical Research Council (2017). UK-Wide Survey of Clinical and Health Research Fellowships.

[16] Medical Schools Council (2017). Survey of Medical Clinical Academic Staffinh Levels 2017.

[17] Elo S, Kyngas H (2008). The qualitative content analysis process. J Adv Nurs.

[18] Graneheim U, Lundman B (2004). Qualitative content analysis in nursing research: concepts, procedures and measures to achieve trustworthiness. Nurse Educ Today.

[19] Elo S, Kääriäinen M, Kanste O, Pölkki T, Utriainen K, Kyngäs H (2014). Qualitative Content Analysis: A Focus on Trustworthiness. SAGE Open.

[20] Royal College of Nursing (2020). The nursing and midwifery professoriate in the UK.

[21] Richardson A, Avery M, Westwood G. A cross-funder survey of enablers and barriers to progressing a research-related academic career in the non-medical health professions. https://www.hee.nhs.uk. Accessed Mar 2021.

[22] Trusson D, Rowley E, Bramley L (2019). A mixed-methods study of challenges and benefits of clinical academic careers for nurses, midwives and allied health professionals. BMJ Open.

[23] Cooper J, Mitchell K, Richardson A, Bramley L (2019). Developing the role of the clinical academic nurse, midwife and allied health professional in healthcare Organisations. Int J Pract Based Learn Health Soc Care.

[24] Andreassen P, Christensen MK (2018). “We’re at a watershed”: the positioning of PhD nurses in clinical practice. J Adv Nurs.

[25] Westwood G, Richardson A, Latter S, Macleod Clark J (2018). Fader M building clinical academic leadership capacity: sustainability through partnership. J Res Nurs.

[26] Carrick-Sen D, Richardson A, Moore A, Dolan S (2016). Transforming healthcare through clinical academic roles in nursing, midwifery and allied health professions. A practice resources for healthcare provider organisations.

[27] Strickland K (2017). Developing an infrastructure to support clinical academic careers. Br J Nurs.

[28] Powers J (2020). Increasing capacity for nursing research in magnet-designated organizations to promote nursing research. Appl Nurs Res.

[29] Johantgen M, Weiss M, Lundmark V, Newhouse R, Haller K, Unruh L (2017). Building research infrastructure in magnet® Hospitals: Current status and future directions. JONA.

[30] Berger J, Polivka B (2015). Advancing nursing research in Hospitals through collaboration, empowerment, and mentoring. JONA.

[31] American Nurses Association. ANCC Magnet recognition program. Available from: https://www.nursingworld.org/organizational-programs/magnet/. Accessed Mar 2021.

[32] Kelly LA, McHugh MD, Aiken LH (2012). Nurse outcomes in magnet® and non-magnet hospitals. J Nurs Admin.

[33] Kutney-Lee A, Stimpfel AW, Sloane DM, Cimiotti JP, Quinn LW, Aiken LH (2015). Changes in patient and nurse outcomes associated with magnet hospital recognition. Med Care.

[34] dit Dariel OP and Regnaux JP. Do Magnet®-accredited hospitals show improvements in nurse and patient outcomes compared to non-Magnet hospitals: a systematic review. JBI Evid Synthesis, 2015. 13(6):168–219. 10.11124/jbisrir-2015-2262 26455752

[35] Magnet 4 Europe (2020). Magnet 4 Europe: Improving mental health and wellbeing in the health care workplace.

[36] Royal College of Nursing (2018). Left to chance: the health and care nursing workforce supply in England.

[37] Carrick-Sen DM, Moore A (2019). Improving care and outcome through NMAHP research-focused clinical academic roles – an international perspective. Int J Pract Based Learn Health Soc Care.

[38] Staffileno BA, Wideman M, Carlson E (2013). The financial and clinical benefits of a hospital-based PhD nurse researcher. Nurs Econ.

[39] Wood MD, Powers J, Rechter JL (2019). Comparative evaluation of chest tube insertion site dressings: a randomized controlled trial. Am J Crit Care.

[40] Bramley L, Manning JC, Cooper J (2018). Engaging and developing front-line clinical nurses to drive care excellence: evaluating the chief nurse excellence in care junior fellowship initiativ*e*. J Res Nurs.

[41] Wielenga JM, Tume LN, Latour JM, van den Hoogen A (2015). European neonatal intensive care nursing research priorities: an e-Delphi study. Arch Dis Child Fetal Neonatal Ed.

[42] Broom M, Wainwright L, Spence K, Harris DL, van den Hoogen A (2020). Global neonatal nurses identify research priorities for improving neonatal outcome. J Neonatal Nurs.

[43] Rice M, Davis SL, Soistmann HC, Johnson AH, Gray L, Turner-Henson A (2020). Challenges and strategies of early career nurse scientists when the traditional postdoctoral fellowship is not an option. J Prof Nurs.

[44] Massey D, Ion R, Jackson D (2019). Top tips when starting a career in academic nursing. J Clin Nurs.

