# Supplementary material for: Neonatal nursing led research activity in the UK: a survey of current practice
Source: BMC Nurs. 2021 Oct 18;20:201. doi: 10.1186/s12912-021-00719-8 (PMC8522152; doi:10.1186/s12912-021-00719-8)
Supplement: Supplementary file 1 — Additional file 1: Supplementary Table 1. Participants perceived barriers and facilitators to research and interest in forming neonatal nursing specialist research group [file 12912_2021_719_MOESM1_ESM.docx]

Supplementary table 1: participants perceived barriers and facilitators to research and interest in forming neonatal nursing specialist research group

| **Question** | **Theme** | **Quote** |
| --- | --- | --- |
| Barriers to research | Time | Time is a great barrier - all my clinical duties demand my time. |
|  |  | Major barrier is time. My role is 100% clinical with no time allocated for research or education. |
|  |  | Time- fitting the research around a full time post even though research is considered part of my role |
|  |  | Time is also an issue for 'shop floor' staff as they cannot be released to spend specific time on projects |
|  | Knowledge of / access to funding | Funding - there is a lack of funding and poor advertising of funding opportunities that are available outside the research environment - I was totally unaware of potential funding opportunities until I was working in research |
|  |  | There is a lack of funding to facilitate research in the clinical setting for nurses…Research opportunities are not widely known by the nursing staff |
|  |  | I would have liked to do PhD but no funding |
|  | Colleague apathy | Motivation of team to be involved in research- variable |
|  |  | Staff engagement presents an issue |
|  |  | Lack of interest from colleagues |
|  |  | Perception of nursing research and motivation to lead research - although research is a 'pillar' of advanced practice, I have found that through conversation and experience, even fellow ANNPs are not currently motivated to lead their own research projects |
|  |  | If nurses would lead research within their own team, the team would be more involved. |
|  | Lack of role models | (lack of)…availability of experienced neonatal researchers to support your work. |
|  |  | (lack of)…mentoring support |
|  |  | neonatal nurses may need more role models in the profession or experience of research |
|  |  | Lack of collaborators with neonatal PhD |
|  | Limited nursing research knowledge | There is a lack of education within the nursing force in regards to research and it’s facilitation |
|  |  | not all nurses have knowledge about the research process |
|  |  | There is a lot of mystery surrounding research |
|  | Limited priority | conflicting demands take up time away from research as it becomes less of a priority. |
|  |  | neonatal nurse research not high priority for funding bodies |
|  | Confusion over nursing role | We have two research nurses…but they spend their time running after the medical staff and the studies that they PI for, not on any nursing research. The funding is from the NIHR and so this limits what they are supposed to be doing in their role. Nursing research and the NIHR roles are incompatible with moving this agenda on in my view. |
|  |  | We have a team of research nurses who help data collect, consent e.t.c. but we do not have 'nurse researchers'. |
| Facilitators | Support | The support of Consultants in pursuing this role has been hugely beneficial in achieving funding and developing an academic role for me. |
|  |  | Commitment from the trust to Consultant Nurse posts have 50% non-patient facing time to undertake research |
|  |  | (a) dedicated research nurse who is able to support the staff on the front line undertaking the practical aspects of studies |
|  | Research culture | the presence of the (research) team raises the profile of research and its relevance to clinical practice, which could help to encourage potential nurse researchers of the future. |
|  |  | Workplace culture of research |
|  | Role | As a clinical research nurse, I personally find the balance really good. |
| Other comments | Advancing neonatal nursing career options | Neonatal nurses need more options to progress and research is definitely one option…being experts in neonatal care neonatal nurses are ideally situated to lead on the development of neonatal research to improve the care and practices surrounding neonatal care. |
|  |  | For the future of neonatal nursing I strongly believe that there needs to be a change in culture in accepting other advanced nursing roles in neonatal care, in particular in education roles. We really do need to think outside the box if this is to be widely recognised as a means to future role development. |
|  |  | For nurses that would like to advance their academic and research skills but not necessarily pursue the ANNP route, Nurse Researcher roles within neonates could be developed |
|  | Role modelling | The future for neonatal research should include nurse led research centres |
|  |  | Lack of collaboration between research active neonatal nurses within UK. |
|  | Essential education | Higher degrees are essential in a specialized area of nursing such as neonates. This area of nursing is continuously fast developing and it is important the staff are too. |
|  |  | Vital role for continued developments within the NHS |
| Interested in forming neonatal nurses group |  |  |
| - yes | Raise profile of nursing | We should be advancing our role to become specialist research practitioners. |
|  |  | Important to raise the profile of research and within neonatal nursing |
|  |  | Together we could do much more and create powerful body in the neonatal field. |
|  | Collaboration | A network which is providing support, challenging ideas and enhances the possibility of nurse led research would be amazing. |
|  |  | We need to work together to drive Neonatal nurse involvement in research forward. |
|  |  | Collaboration is the key to developing high quality translatable research; inspiring other nurses that this is a career option for them. |
|  | Personal development | This...may provide me with the confidence and knowledge to undertake more research |
|  | Future specific supervisors | My academic supervisors for my masters were both midwives as they were the people who had the closet link with neonatal practice. I'd like to think in future perhaps neonatal nurses like myself would be able to supervise and supports others doing neonatal research. |
| - *no* | Time / experience | Do not feel I have the experience or expertise required |
|  |  | I doubt very much if my employer would allow me to do this in work time |
